# Experimental and numerical studies on heat exchange between slag/metal particles and air inside countercurrent cyclone heat exchanger for recovering waste heat from molten slags in dry and centrifugal granulation process

**DOI:** 10.1371/journal.pone.0349252

**Published:** 2026-07-23

**Authors:** Shali Lv, Yuhua Pan, Yansong Li, Ming Zhao, Yu-an Jing, Changyou Cai, Jiaqi Zhong, Jun Wang, Haixin Guo

**Affiliations:** 1 School of Materials and Metallurgy, University of Science and Technology Liaoning, Anshan, China; 2 Hot Rolled Strip Steel Mill, Ansteel Co. Ltd., Anshan, China; 3 School of Electronic and Information Engineering, University of Science and Technology Liaoning, Anshan, China; SASTRA Deemed University, INDIA

## Abstract

In dry and centrifugal granulation of molten slags with heat recovery, the heat exchange efficiency between high-temperature particles and the cold air directly determines the efficiency of waste heat recovery. However, the heat transfer characteristics between pure slag particles granulated from blast furnace slag and air and between a mixture of slag and metal particles granulated from steelmaking slag and air in complex flow fields still remain unclear. Therefore, this study employs a combined approach of experiment and numerical simulation to comparatively investigate the heat transfer behavior of pure slag particles and mixtures of slag and metal particles in a countercurrent cyclone heat exchanger. The investigation results indicate that the heat exchanger exhibits optimal heat transfer performance under specific operating conditions, with the maximum heat exchange efficiencies being 80.0% for pure slag particles and 77.0% for a mixture of slag/metal particles produced from steelmaking slag. Both experimental and numerical simulation results indicate that metallic iron particles produced from granulation of steelmaking slag lowers the heat exchange efficiency; when the metallic iron content in the slag increases from 2.5% to 10% by weight, the heat exchange efficiency drops from 77.0% to 73.0%. The numerical simulation results further reveal that the spatial distribution of particles within the heat exchanger is a key factor determining the overall heat exchange efficiency. The results provide a theoretical basis for design and operation optimization of industrial countercurrent cyclone heat exchangers for waste heat recovery from molten slags in dry and centrifugal granulation process.

## 1. Introduction

The iron and steel industry is currently facing the dual pressures of high energy consumption and environmental pollution [1]. High-temperature molten slag, such as blast furnace ironmaking slag and steelmaking slag (blast furnace slag and steel slag for short, respectively) is the primary by-product of these processes and a key contributor to the energy and environmental challenges mentioned above. Relevant data indicate that the discharge temperature of blast furnace slag ranges from 1450°C to 1550°C, with an annual production of approximately 300 million tons in China. The sensible heat contained in blast furnace slag is estimated to be 1.26–1.88 GJ/t [2,3]. For steel slag, the discharge temperature ranges from 1400°C to 1600°C, with a production volume accounting for approximately 10%−15% of crude steel output [4], and its sensible heat resources are about 1.2–1.5 GJ/t. However, due to current technological limitations, the sensible heat recovery rate for blast furnace slag remains low (typically below 20%) [5], and the comprehensive utilization rate of steel slag remains below 30% [6], and virtually no heat is recovered from the steel slag. Recently, blast furnace slag has also been explored for advanced applications beyond traditional building materials. For instance, slag-derived layered double oxides have been developed as effective CO_2_ adsorbents [7]. Kinetic and modeling studies have further deepened the understanding of CO_2_ adsorption mechanisms in slag-derived materials [8]. The widespread use of traditional treatment processes, such as water quenching and hot splashing, not only wastes substantial high-grade waste heat [9] but also consumes significant water resources and poses environmental pollution risks [10]. Therefore, overcoming the limitations of traditional processes and developing efficient, clean technologies for waste heat recovery from molten slag is of strategic importance for promoting the green and low-carbon transformation of the iron and steel industry [11,12].

To achieve coordinated energy and resource recovery from molten slag, dry and centrifugal granulation technology has become a research hotspot due to its ability to simultaneously produce high-quality slag particles and recover waste heat [13]. Existing studies have primarily investigated the effects of structural configurations (such as spinning cups and discs) and operating parameters on the granulation characteristics of molten slag. Through combined experimental and numerical simulation approaches, these studies have elucidated the dynamic mechanisms of liquid film formation and breakup [14–19]. However, efficient granulation is only the first step in waste heat recovery. The subsequent core challenge lies in efficiently and controllably recovering the heat contained within the high-temperature slag particles. This remains the critical obstacle hindering the breakthrough of this technology for industrial application [20].

Regarding the cooling and heat transfer process of initially semi-solidified slag particles formed by centrifugal granulation, existing research exhibits certain shortcomings. At the mechanistic level, current numerical simulations are mostly confined to the phase change solidification and cooling of single particles, failing to consider the macroscopic flow, distribution, and synergistic heat transfer effects of the particles in actual engineering applications. This creates a disconnect between microscopic mechanism research and macroscopic performance analysis. At the methodological level, the optimization of process parameters is constrained by either high-cost high-temperature experiments or simplified empirical models. Currently, there is a lack of reliable methods or models that can accurately describe the heat exchange behavior between the hot particles and the coolant (commonly cold air).

In response to the above-mentioned issues, dry and centrifugal granulation technology has emerged [13]. As a representative next-generation molten slag treatment technology, this process uses a high-speed rotating disk or cup to break molten slag into droplets, simultaneously producing solid slag particles and recovering waste heat through air cooling [21]. The feasibility of this technology has been verified through experimental and pilot-scale studies. Pickering et al. [22] constructed a rotary cup centrifugal granulation experimental platform. Pilot-scale industrial researches conducted by the Commonwealth Scientific and Industrial Research Organisation (CSIRO) [14] in Australia and by CISDI Thermal & Environmental Engineering Co., Ltd. in China further confirmed its potential for industrial application [21,23]. Extensive studies have been conducted on centrifugal granulation of molten slag using rotating disks and cups, covering liquid film dynamics, droplet breakup, and initial particle solidification [24–28]. However, these studies focus primarily on the granulation step rather than the subsequent gas–solid heat transfer in a dedicated heat exchanger.

Nevertheless, while researches that have so far been conducted and reported in literature mostly focus on spinning disk/cup performance and resultant characteristics of granulated slag particles,the coupling mechanisms among particle swarm motion, spatial distribution, and gas-solid heat transfer within the countercurrent cyclone heat exchanger remain poorly understood. Relatively little attention has been paid to effective cooling of the initially semi-solidified particles and the efficiency of heat exchange between those particles with cold air, which is commonly used as the major coolant. The heat exchange between the hot particles and air is nearly equally important to the centrifugal granulation step, determining the feasibility of the whole process. Therefore, a novel countercurrent cyclone heat exchanger is proposed by the authors that is specially designed for recovering waste heat both from the pure slag particles produced from granulation of molten blast furnace slag and from the mixtures of slag and metal particles produced from granulation of metallic iron-bearing molten steel slag. By taking such countercurrent cyclone heat exchanger being the research object, this study focuses on the characteristics of heat exchange between the slag/metal particles and the cooling air, with the aim to quantitatively reveal how structural and operating parameters of the heat exchanger influence the heat transfer performance of the system through performing both the modeling experiments and the CFD numerical simulations, so as to establish the optimal structural and operating conditions for efficient and stable operation of the heat exchanger. It is expected that the present research results not only validate the technical feasibility of the countercurrent cyclone heat exchanger to be used as molten slag waste heat recovery device but also provide critical data for constructing high-precision heat transfer models for such devices, thereby laying a theoretical foundation for subsequent industrial scale-up and engineering applications. Moreover, compared to the conventional water quenching process, the proposed method eliminates water consumption, avoids the generation of harmful pollutants and fugitive dust, and significantly reduces the associated environmental burdens, making it a more sustainable alternative for slag treatment.

## 2. Experiment

To ensure the rigor and reliability of the study, the following sections describe a combined approach of experimental measurement and numerical simulation by first elaborating on the material properties and then introducing in detail the experimental design and the numerical simulation.

### 2.1. Material properties

The physical properties of blast furnace slag particles and metallic iron particles used in this study are detailed in Table 1 [29]. The main chemical compositions of the slag are (in mass percentage): 43.45%CaO, 34.35%SiO_2_, 13.02%Al_2_O_3_, 5.83%MgO, and 0.15%MnO. Due to the complexity of the heat transfer process, and to simplify the calculations and improve simulation efficiency, the physical property parameters of air and particles are set as fixed values.

**Table 1:** Physical properties of materials.

Parameters	Unit	Air	Slag particles	Metallic iron particles
Density	kg/m^3^	1.225	3100	8030
Specific heat capacity	J/(kg·K)	1006.43	1680	502.49
Thermal conductivity	W/(m·K)	0.0242	–	–
Viscosity	kg/(m·s)	1.7894 × 10^−5^		–
Emissivity	–	–	0.85	0.7

### 2.2. Experimental Set-up

**Fig 1:**
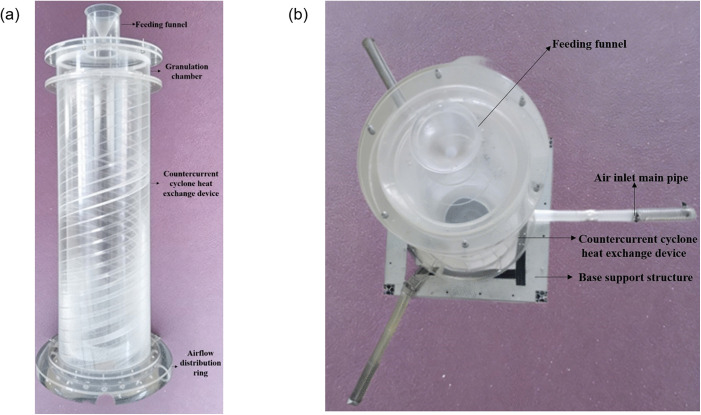
Photo of experimental set-up. (a) Sideview; (b) Top view.

**Fig 2:**
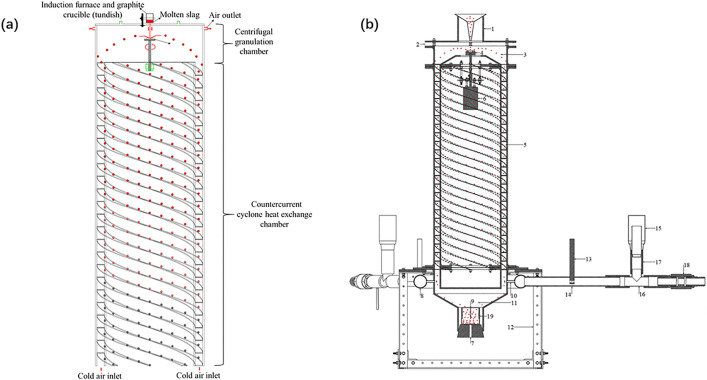
Schematic diagram of experimental set-up. (a) Prototype; (b) Experimental physical model.

Due to challenges such as the high-temperature requirements for experimental equipment and the limitations of visual observation in actual high-temperature experiments on centrifugal granulation of molten slag with waste heat recovery, in this study a low-temperature countercurrent cyclone heat exchange experimental device was constructed in laboratory based on the similarity principle [30,31]. The overall structure of this experimental heat exchanger is shown in Figs 1 and 2. The basic idea of the present approach is to use rising hot air to heat up the falling cold particles, which is an inverse process of the real situation where the falling hot particles are cooled by the rising cold air. The research adopted a technical pathway of experiment first, followed by numerical simulation validation, by equivalently converting the cooling process of high-temperature particles under actual operating conditions into a countercurrent heat exchange process where hot air heats room-temperature particles. To demonstrate the equivalence between the low-temperature inverse experiment and the high-temperature prototype, we evaluated the key dimensionless numbers based on the principle of similarity. The Prandtl numbers are 0.67 for both cases, confirming good similarity in fluid properties. The Nusselt numbers are 4.25 and 3.3 for the low-temperature and high-temperature conditions, respectively. This deviation primarily arises from differences between the idealized simulation conditions and the experimental setup, including wall heat losses and measurement accuracy. However, the deviation is within the acceptable range for engineering similarity analysis, and the low-temperature experiment is considered capable of reflecting the gas‑solid heat transfer behavior of the high-temperature prototype. To ensure the accuracy of experimental data, three hot air blowers were arranged circumferentially at 120° angle intervals to deliver hot air evenly into the device from its bottom and for this an airflow distribution ring was installed at the bottom, effectively ensuring the uniformity of hot air supply to the heat exchanger.

During the experimental preparation stage, the slag and slag/metal particle mixtures were first weighed. Hot air blowers were activated to introduce hot air into the countercurrent cyclone heat exchanger, and the airflow rate was precisely adjusted using flow controllers. After monitoring with thermocouples to ensure that the air temperature at the inlet and inside the device reached the preset values and remained stable, the rotary cup was adjusted to the target rotational speed, and the initial temperature of the slag was recorded. Subsequently, the experiment commenced. During the experiment, the slag particles that fell through the funnel onto the rotary cup were thrown into the granulation chamber under the centrifugal force, and entered the swirl channels after colliding with the wall of the granulator. The slag particles underwent multiple collisions and heat exchange with rising hot air and with the wall surface inside the channel, finally falling into the particle collection tank. Their average outlet temperature was measured using pre-installed thermocouples inside the tank.

### 2.3. Experimental scheme

The experimental schemes designed in the present work are given in Tables 2–5, which investigate the effects of particle flow rate, hot air flow rate, particle size, and metallic iron content on the flow and heat transfer behavior of slag particles and slag-metal particle mixtures inside the experimental countercurrent cyclone heat exchanger. In Tables 2 and 4, the hot air flow rate and the particle flow rate were increased synchronously, whose relationship was derived from heat exchange balance based on designed inflow and outflow temperatures, to examine the combined effect of gas and solid flow rates on heat transfer performance, following the common experimental practice in gas-solid countercurrent heat transfer studies [32]. Each experimental condition was independently repeated three times to ensure the reproducibility of the experimental results. The average values of the outlet particle temperature, outlet air temperature, and heat transfer efficiency were obtained from repeated experiments. The relative deviation between repeated experiments is controlled within 2%, indicating that the experiment has good stability and repeatability.

**Table 2:** Experimental scheme for investigating effect of slag flow rate variation.

Scheme	Rotary cup speed (RPM)	Slag flow rate (kg/min)	Slag particle size (mm)	Hot air flow rate (m³/h)
1	2100	0.36	0.6-0.88	20
2	2100	0.42	0.6-0.88	26
3	2100	0.48	0.6-0.88	32
4	2100	0.54	0.6-0.88	38

**Table 3:** Experimental scheme for investigating effect of particle size variation.

Scheme	Rotary cup speed (RPM)	Slag flow rate (kg/min)	Hot air flow Rate (m³/h)	Slag particle size (mm)
1	2100	0.48	32	0.425
2	2100	0.48	32	0.6
3	2100	0.48	32	0.88
4	2100	0.48	32	1.18

**Table 4:** Experimental scheme for investigating effect of slag-metal particle mixture flow rate variation.

Scheme	Rotary cup speed (RPM)	Particle mixture flow rate (kg/min)	Iron content (wt%)	Hot air flow rate (m³/h)
1	2100	0.36	5	20
2	2100	0.42	5	26
3	2100	0.48	5	32
4	2100	0.54	5	38

**Table 5:** Experimental scheme for investigating effect of metallic iron content variation in steel slag.

Scheme	Rotary cup speed (RPM)	Hot air flow rate (m³/h)	Particle mixture flow rate (kg/min)	Iron content (wt%)
1	2100	32	0.48	2.5
2	2100	32	0.48	5
3	2100	32	0.48	7.5
4	2100	32	0.48	10

## 3. Analysis of experimental results

Heat exchange efficiency is defined as the ratio of the actual heat absorbed by the particles to the theoretical maximum heat transfer. It is calculated based on the heat balance between the particles and the air stream using the following equation:


$$\begin{array}{c} Q=cm\Delta T \end{array}$$ (1)

The heat release of fluid 1, Φ_1_ is:


$$\begin{array}{c} \Phi_{1}=q1c1(t_{\text{1}}^{\prime }-t_{\text{1}}^{"}) \end{array}$$ (2)

The heat absorption of fluid 2, Φ_2_ is:


$$\begin{array}{c} \Phi_{2}=q2c2(t_{\text{2}}^{\prime }-t_{\text{2}}^{"}) \end{array}$$ (3)

The heat recovery rate (efficiency) of a heat exchanger is defined as the ratio between the actual heat transferred by the heat exchanger, Φ, and the theoretically maximum possible heat transferred, Φ_max_. In this article, due to the neglect of heat loss, the maximum possible heat transfer is the heat released by fluid 1 (particle phase) Φ_1_, while the actual heat transfer is the heat absorbed by fluid 2 (air) Φ_2_.

Thus, η is defined as


$$\begin{array}{c} \eta =\frac{\Phi_{2}}{\Phi_{1}}=\frac{q2c2(t_{\text{2}}^{\prime }-t_{\text{2}}^{"})}{q1c1(t_{\text{1}}^{\prime }-t_{\text{1}}^{"})} \end{array}$$ (4)

where *q* is the flow rate of the fluid, measured in kg/s; *c* is the specific heat capacity of the fluid in J/(kg·K); *t* is the temperature of the fluid in K.

In this study, Φ_1_ represents the heat released by the air; Φ_2_ is the heat absorbed by the slag.

### 3.1. Heat exchange experiment in countercurrent cyclone heat exchanger on centrifugally granulated blast furnace slag particles

#### 3.1.1. Effect of slag particle flow rate on particle-air heat exchange performance.

**Fig 3:**
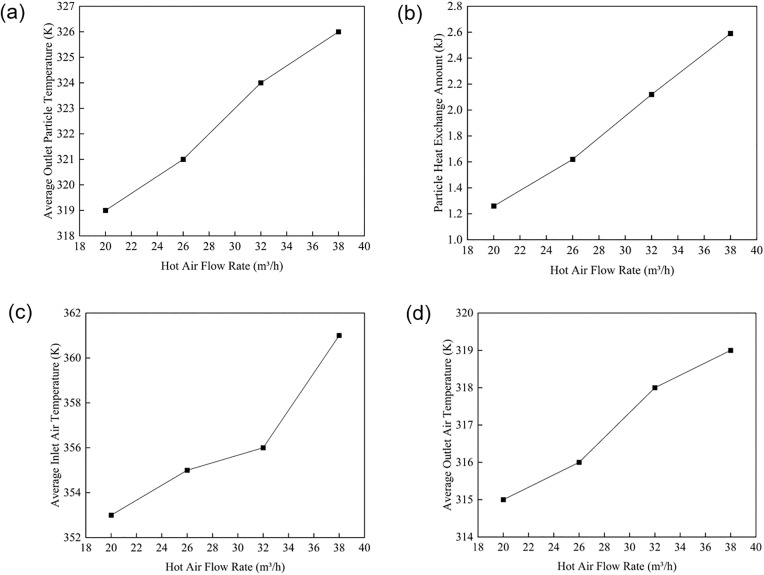
Effect of hot air flow rate on heat exchange performance. (a) Variation of average particle temperature; (b) Variation of heat exchange amount; (c) Variation of average air inlet temperature; (d) Variation of average air outlet temperature.

Fig 3 illustrates the variation pattern of heat exchange performance of the countercurrent cyclone heat exchanger under combined operating conditions (i.e., synchronous changes in cold slag particle and hot air flow rates). The results show that with the synchronous increase in particle and air flow rates, both the slag outlet temperature and the total heat exchange amount increased. This is mainly because the increase in hot air flow rate enhances the intensity of gas-solid convective heat transfer, while the increase in hot air flow rate also increases the total heat entering the system. Table 6 summarizes the heat exchange efficiency of the device under the combined operating conditions, where the rotary cup speed is fixed at 2100 RPM, and the hot air flow rate and slag flow rate are adjusted proportionally based on heat balance calculation. The heat exchange efficiency remains essentially consistent across all tested conditions, with an average value of 47.5% and a limited fluctuation range between 45.8% and 50.0%. This stable performance confirms the rationality of the heat balance-based flow rate matching strategy, ensuring reliable heat transfer under synchronous variation of operating parameters.

**Table 6:** Heat exchange efficiency under different combined operating conditions.

Rotary cup speed (RPM)	Hot air flow rate (m³/h)	Slag particle flow rate (kg/min)	Heat exchange efficiency (%)
2100	20	0.36	49.6
2100	26	0.42	47.9
2100	32	0.48	52.0
2100	38	0.54	48.2

#### 3.1.2. Effect of slag particle size on heat exchange performance.

**Fig 4:**
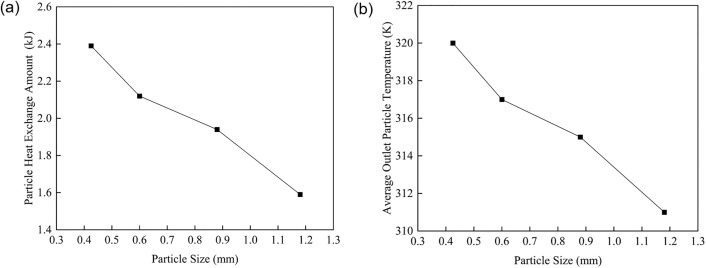
Effect of particle size on heat exchange performance. (a) Variation of particle heat exchange amount; (b) Variation of average outlet particle temperature.

Fig 4 presents a comparison of the heat exchange performance of slag particles with different sizes under identical flow rate conditions. As the particle size increased, both the slag outlet temperature and the total heat exchange amount decreased. The increase in particle size reduces its specific surface area, which diminishes the effective contact area available for heat transfer, thereby weakening the overall heat transfer intensity. Furthermore, the sensitivity of heat transfer to particle size indicates that controlling the particle size distribution during slag granulation and waste heat recovery is important for optimizing heat exchange performance. This particle size-dependent heat exchange characteristic provides a reference for optimizing the target particle size range in the industrial granulation process. Table 7 shows the heat exchange efficiency of the device for different slag particle sizes. As the particle size increased, the heat exchange efficiency decreased significantly, further confirming the critical role of particle size in the gas-solid heat exchange process. It is worth noting that the experimental data presented in this section were obtained from experiments conducted in winter, while the other three sets of experiments were carried out in summer. Despite the seasonal variation in ambient conditions, the same overall trend in heat exchange performance with respect to particle size was consistently observed across all four sets of experiments, thereby further validating the reliability of the experimental results.

**Table 7:** Heat exchange efficiency for different particle sizes.

Rotary cup speed (RPM)	Slag particle flow rate (kg/min)	Hot air flow rate (m³/h)	Particle size (mm)	Heat exchange efficiency (%)
2100	0.48	32	0.425	67.7
2100	0.48	32	0.6	59.8
2100	0.48	32	0.88	54.6
2100	0.48	32	1.18	44.2

### 3.2. Heat exchange experiment in countercurrent cyclone heat exchanger on mixtures of slag and metallic iron particles

#### 3.2.1. Effect of steel slag flow rate on heat exchange performance.

**Fig 5:**
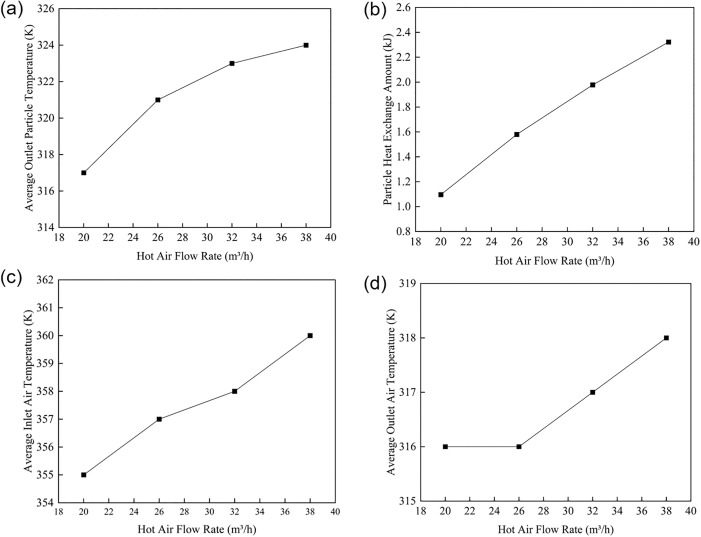
Effect of hot air flow rate on heat exchange performance for slag-metal particle mixtures. (a) Variation of average particle temperature; (b) Variation of particle heat exchange amount; (c) Variation of average air inlet temperature; (d) Variation of average air outlet temperature.

Fig 5 presents the experimental results under combined operating conditions (i.e., synchronous variation of slag-metal particle mixture flow rate and hot air flow rate) for processing steel slag containing 5 wt.% metallic iron. The results show that with the synchronous increase in particle and air flow rates, both the outlet temperature of the slag-metal particle mixture and the total heat exchange amount increased, a trend consistent with that observed for pure blast furnace slag particles under the same conditions. This is mainly because the increase in hot air flow rate enhances the intensity of gas-solid convective heat transfer, while also increasing the total heat entering the system. Table 8 summarizes the heat exchange efficiency of the device under different combined operating conditions. It can be seen from this table that, under all tested conditions, the heat exchange efficiency generally remains stable, with an average value of 43.6% and a narrow fluctuation range between 42.2% and 45.0%, again confirming the rationality of the heat balance-based flow rate matching strategy.

**Table 8:** Heat exchange efficiency for slag-metal particle mixtures (with 5 wt.% metallic rion).

Rotary cup speed (RPM)	Hot air flow rate (m³/h)	Particle mixture flow rate (kg/min)	Heat exchange efficiency (%)
2100	20	0.36	42.2
2100	26	0.42	44.3
2100	32	0.48	45.0
2100	38	0.54	43.4

#### 3.2.2. Effect of metallic iron content on heat exchange performance.

**Fig 6:**
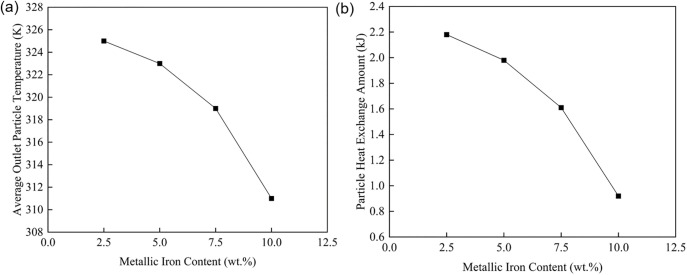
Effect of iron content on heat exchange performance. (a) Variation of average particle temperature; (b) Variation of particle heat exchange amount.

Fig 6 illustrates the effect of metallic iron content in steel slag on the heat exchange behavior of slag-metal particle mixtures under identical flow rate conditions. At an initial temperature of 300 K, both the outlet particle temperature and the total heat exchange amount decreased with increasing metallic iron content, and the magnitude of this decrease increased further with increasing the metallic iron content. This phenomenon can be attributed to two main factors. First, the thermophysical properties of the particles change. Metallic iron generally has a lower heat capacity than slag, and the heat absorption capacity of the particle mixture system is primarily determined by the heat capacity of its components. The increase in iron content reduces the overall heat capacity of the particle mixture, thereby weakening their heat absorption capability. Second, the particle flow characteristics are affected. Due to the significant differences in physical properties such as density between metallic iron and slag, the motion trajectory and velocity distribution of the particle mixture, weakening the intensity of gas-solid convective heat transfer.

Table 9 presents the heat exchange efficiency of the device for different metallic iron contents. The results indicate that the heat exchange efficiency decreases with increasing metallic iron content. When the metallic iron content increased from 2.5% to 10%, the heat exchange efficiency dropped by 28.5%. In the present slag-metal mixture, the reduction in heat exchange efficiency caused by the addition of metallic iron can be attributed to two factors: the lower specific heat capacity of iron and the altered particle trajectories due to its higher density. Based on the present data, the thermophysical effect is considered the dominant factor, as the heat capacity of iron is about one-third of that of slag, leading to a noticeable reduction in the overall heat absorption capacity of the particle mixture. In contrast, the hydrodynamic effect is weaker: although iron particles migrate more rapidly toward the wall due to higher centrifugal force, the resulting change in the gas-solid contact pattern has a relatively minor impact on the overall heat exchange efficiency under the present operating conditions.

**Table 9:** Heat exchange efficiency for different metallic iron contents.

Rotary cup speed (RPM)	Slag-metal particle mixture flow rate (kg/min)	Metallic iron content (%)	Heat exchange efficiency (%)
2100	0.48	2.5	47.8
2100	0.48	5	44.0
2100	0.48	7.5	35.5
2100	0.48	10	20.1

## 4. Numerical simulation on experimental countercurrent cyclone heat exchanger

### 4.1. Computation domain

**Fig 7:**
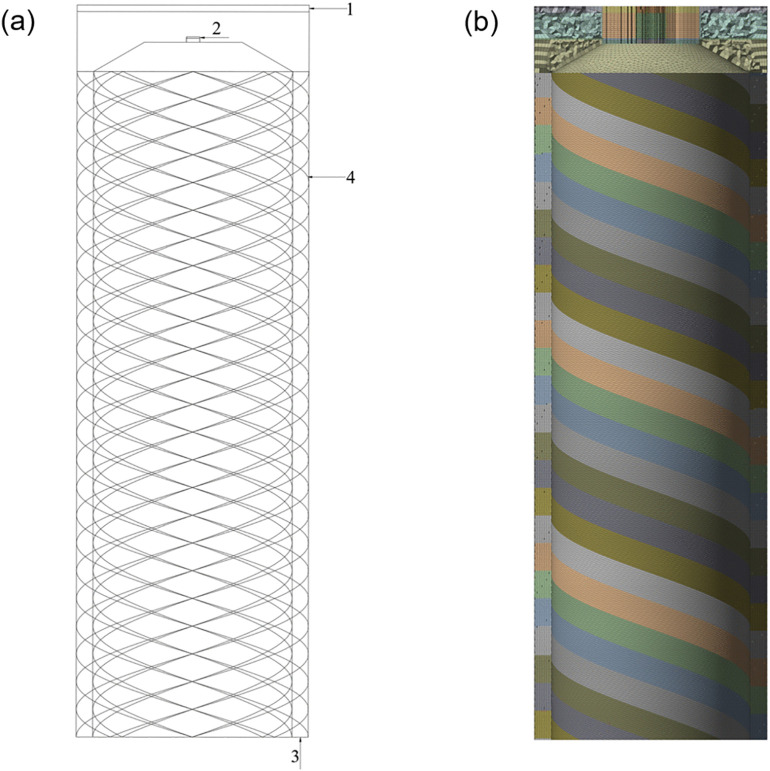
Experimental setup and computational mesh. (a) Geometric model of the experimental setup. (b) Diagram of the computational mesh.

As shown in Fig 7(a), the physical model dimensions used in the simulation were the same as those of the experimental setup (1:1 scale). The diameter and height of the experimental countercurrent cyclone heat exchange device are 300 mm and 943.35 mm, respectively; the height of the side annular hot air outlet is 8.6 mm; Totally 8 swirl vanes were installed in the heat exchange chamber forming 8 swirl channels for heat exchange, each with a width of 21.4 mm. Fig 7(b) also shows the schematic of the computational mesh.

### 4.2. Numerical model assumptions

This study primarily investigates the motion and cooling processes of high temperature particles within the granulation chamber and the heat exchange chamber. Based on the following fundamental assumptions, a three-dimensional computational fluid dynamics (CFD) numerical model was established to explore the motion behavior and heat transfer characteristics of particle groups within the granulation chamber and the heat exchange chamber:

(1) The particles (both slag and metal) are assumed to be standard spheres with uniform particle size, maintaining their shape unchanged during the motion and heat transfer process, and heat conduction within the particles is neglected.
(2) The phase change phenomenon (e.g., crystallization) inside the particles during the cooling process is neglected.
(3) Air is assumed to be an incompressible fluid.

Under high concentration particle flow conditions, the contact heat conduction between particles and between particles and the wall may have an impact on the overall heat transfer process. However, under the working conditions of this study, at the same location the collided particles would have very small temperature difference and thus limited heat exchange among them that would eventually be transferred to air. In addition, the particles mainly exhibit swirling motion and have a short duration of contact with the heat exchanger wall. Therefore, gas-solid convective heat transfer dominates. Based on model simplification and computational efficiency considerations, we neglected the contact heat conduction between particles and between particles and walls.

### 4.3. Governing equations

#### 4.3.1. Governing equations for the solid phase.

(1) Analysis of Forces Acting on Particles

The particle diameters investigated in this study are 0.65 mm for slag and 1 mm for metallic iron. As these sizes are all below the criteria for defining submicron particles [33] that typically refer to particles with diameters being less than 1 μm. Therefore, only the gas-phase drag force on particles, gravity, and collision forces between particles are considered in the present numerical simulation. Based on Newton’s second law, the equation for motion of particles in the Cartesian coordinate system can be expressed as:


$$\begin{array}{c} m_{p}\frac{du_{p}}{dt}=FG+Fi+Fd \end{array}$$ (5)

where: *m*_*p*_ is the mass of a particle, kg; ***u***_*p*_ the particle velocity, m/s; *t* the time, s; ***F***_*G*_ the gravitational force acting on the particle, N; ***F***_*i*_ the collision contact force between particles, N; and ***F***_*D*_ the drag force exerted by the gas phase on the particle, N.

(2) Drag Force Model

In this study, particles are considered as standard spheres. Therefore, when analyzing the force exerted by air on the particles, the Spherical Drag Model was adopted, as expressed by Eq. (2) [34]:


$$\begin{array}{c} FD,0=CD\frac{\text{1}}{\text{2}}\rho {u_{p}}^{\text{2}}\frac{\pi {dp}^{\text{2}}}{\text{4}} \end{array}$$ (6)

where *C*_*D*_ is the drag coefficient; *d*_*p*_ the diameter of a standard spherical particle with the same volume as the actual particle and *ρ* the fluid density, kg/m³. Considering the influence of surrounding particles on the drag force, Di modified the above formula as [35]


$$\begin{array}{c} F_{D}=F_{D,0}\varepsilon^{-\chi } \end{array}$$ (7)

$$\begin{array}{c} \chi =3.7-0.65exp\left[-\frac{{\left(1.5-lgR_{N}\right)}^{\text{2}}}{\text{2}}\right] \end{array}$$ (8)

$$\begin{array}{c} R_{N}=\frac{\varepsilon \rho \left|ug-up\right|dp}{\mu g} \end{array}$$ (9)

where *R*_*N*_ is the Reynolds number; ***u***_*g*_ the fluid velocity, m/s; *𝜇*_*g*_ the dynamic viscosity of the fluid, Pa·s; *χ* the empirical correction coefficient.

In Eq. (9), the grid porosity, *ε*, can generally be calculated as follows


$$\begin{array}{c} \varepsilon =1-\sum_{i=1}^{np}\frac{vp,i}{vcell}, \end{array}$$ (10)

where *n*_*p*_ represents the total number of particles in the CFD grid cell; *v*_*p,i*_ the volume of the particle *i*, m³; *v*_*cell*_ the volume of the grid cell, m³.

Additionally, *C*_*D*_ can be calculated using the following equation


$$\begin{array}{c} CD=\frac{K_{\text{1}}}{R_{N}}+\frac{K_{\text{2}}}{R_{N}^{\text{2}}}+K_{3} \end{array}$$ (11)

where *K*_*1*_, *K*_*2*_ and *K*_*3*_ are derived from the results obtained by Morsi and Alexander [36].

(3) Magnus Force Equation

The expression for the Magnus force *F*_*M*_ is:


$$\begin{array}{c} FM=\frac{\pi }{\text{8}}{dP}^{\text{2}}CRL\rho \frac{\left|V\right|}{\left|\Omega \right|}\left(V\times \Omega \right) \end{array}$$ (12)

where *A*_*p*_ is the projected surface area of the particle, m²; ***V*** the fluid-particle relative velocity, m/s; ***Ω*** the fluid-particle relative angular velocity, rad/s; *C*_*RL*_ the rotational lift coefficient, which is a function of the spin parameter *T*. *C*_*RL*_ is defined as


$$\begin{array}{c} CRL=\left\{ \begin{array}{c} @l@0.4\text{S}\geq 1\\ (0.4\pm 0.1)S\text{S}<1 \end{array}\right. \end{array}$$ (13)

where *S* is the spin parameter, defined as follows


$$\begin{array}{c} S=\frac{\left|\vec{\omega }p\right|dp}{2\left|\vec{u}g-\vec{u}p\right|} \end{array}$$ (14)

in which ***ω***_***p***_ is the angular velocity of the particle, rad/s.

(4) Particle-air Heat Exchange Equation

Convective heat transfer between the particle and air is calculated using


$$\begin{array}{c} Q_{i,f}=h_{cov}A_{i}\Delta T_{m} \end{array}$$ (15)

where *h*_*cov*_ is the convective heat transfer coefficient, W/(m²·K); *A*_*i*_ the surface area of the particle *i*, m²; and *∆T*_*m*_ the temperature difference between the particle and the air, K.

Radiative heat transfer between the particle and the wall is given by:


$$\begin{array}{c} Q_{i,rad}=\sigma \varepsilon_{i}A_{i}\left(T_{i}^{\text{4}}-T_{local}^{\text{4}}\right) \end{array}$$ (16)

where *σ* is the Stefan-Boltzmann constant, W/(m²·K⁴); *ε*_*i*_ the emissivity of particle *i*; *T*_*i*_ the temperature of particle *i*, K; and *T*_*local*_ the average temperature of particles within a grid cell, K.

#### 4.3.2. Governing equations for the fluid phase.

The fluid flow process can be described by the following system of partial differential equations:

(1) Mass Conservation Equation (Continuity Equation):


$$\begin{array}{c} \frac{\partial \left(\rho u_{i}\right)}{\partial x_{i}}=0 \end{array}$$ (17)

where *u*_*i*_ represents a component of the gas-phase velocity in the Cartesian coordinate direction, m/s and *x*_*i*_ a component of the Cartesian coordinate system, m.

(2) Momentum Conservation Equation:


$$\begin{array}{c} \frac{\partial \left(\rho u_{i}u_{j}\right)}{\partial x_{j}}=-\frac{\partial p}{\partial x_{i}}+\frac{\partial \left(\tau_{ij}\right)}{\partial x_{j}}+\rho g_{i}+f \end{array}$$ (18)

where *p* is the static pressure, Pa; *τ*_*ij*_ the stress tensor (i.e., the components of the viscous stress on an infinitesimal element surface); and *ρg*_*i*_ and *f* are, respectively, the components of gravitational body force and external body force (such as forces arising from interaction with the particle phase) in the *i*th-direction, as well as other source terms, N/m³.

(3) Realizable *k-ε* Turbulence Equations:

$$\begin{array}{c} \frac{\partial }{\partial x_{j}}\left(\rho ku_{j}\right)=\frac{\partial }{\partial x_{j}}\left[\left(\mu +\frac{\mu_{t}}{\sigma_{k}}\right)\frac{\partial k}{\partial x_{j}}\right]+G_{k}+G_{b}-\rho \varepsilon -Y_{M}+S_{k} \end{array}$$ (19)

$$\begin{array}{c} \frac{\partial }{\partial xj}\left(\rho \varepsilon uj\right)=\frac{\partial }{\partial xj}\left[\left(\mu +\frac{\mu_{t}}{\sigma_{\varepsilon }}\right)\frac{\partial \varepsilon }{\partial xj}\right]+\rho C_{\text{1}}S_{\varepsilon }-\rho C_{\text{2}}\frac{\varepsilon^{\text{2}}}{k+\sqrt{v\varepsilon }}+C_{1\varepsilon }\frac{\varepsilon }{k}C_{3\varepsilon }G_{b}+S_{\varepsilon } \end{array}$$ (20)

where


$$\begin{array}{c} C_{\text{1}}=max[0.43,\frac{\eta }{\eta +5}],\eta =S\frac{k}{\varepsilon },S=\sqrt{2S_{ij}S_{ij}} \end{array}$$ (21)

In the above equations, *G*_*k*_ represents the generation term of turbulent kinetic energy due to mean velocity gradients, (W.kg/m^3^); *G*_*b*_ the generation term of turbulent kinetic energy due to buoyancy, (W.kg/m^3^); *Y*_*M*_ the contribution of fluctuating dilatation in compressible turbulence to the overall dissipation rate, (W.kg/m^3^); *μ*_*t*_ the turbulent viscosity, Pa·s; *k* the turbulent kinetic energy, m²/s²; *ε* the turbulent dissipation rate, m²/s³; *C*_*2*_, *C*_*1ε*_, *C*_*3ε*_ are constants; *σ*_*k*_ and *σ*_*ε*_ the turbulent Prandtl numbers for *k* and *ε*, respectively; *S*_*k*_ and *S*_*ε*_ the user-defined source terms; and *S*_*ij*_ the mean strain rate, 1/s.

(4) Energy Conservation Equation:

$$\begin{array}{c} \rho c_{P}u_{j}\frac{\partial T}{\partial x_{j}}=\tau_{ij}\frac{\partial u_{i}}{\partial x_{j}}+\lambda \frac{\partial^{\text{2}}T}{\partial x_{j}^{\text{2}}}+q_{v} \end{array}$$ (22)

where *c*_*p*_ is the specific heat capacity of the fluid at constant pressure, J/(kg·K); λ the thermal conductivity of the fluid, W/(m·K); *q*_*v*_ the heat exchange term between particles and the fluid.

### 4.4. Boundary conditions

Based on the boundary condition values set in Table 10, the particle phase inlet of the model adopts the velocity inlet condition, and the cold air outlet is set as a pressure outlet. The hot air inlet located at the bottom of the model also serves as the particle outlet and is set as a velocity inlet to ensure that particles pass through this outlet into the particle collection tank. Additionally, to simulate the actual operating conditions within the cyclone heat exchanger, a corresponding heat source power was applied to the walls during the simulation to compensate for heat losses occurring in the actual heat exchange process.

**Table 10:** Boundary conditions.

Boundary number and name	Fluid phase	Particles
Hot air inlet/ Heated particle outlet	Velocity inlet	Escape
Cooled air outlet	Pressure outlet	Escape
Cold particle inlet	Velocity inlet	Escape
Walls	–	Reflect

In the present numerical simulation process, we reasonably simplified the actual structure by omitting the geometric details of the rotary cup and replacing it with an array of 36 particle inlets along the periphery of the cup. That is, this annular surface was uniformly divided into 36 independent inlets, with one inlet set every 10°, to simulate the continuous supply of particles into the computational domain. The information for each particle is described by nine basic attribute parameters, including position coordinates, velocity vector, particle diameter, and particle temperature. This simplified model significantly improves computational efficiency while ensuring the accuracy of key physical processes.

In the numerical model, the motion and heat transfer behavior of each particle (or particle parcel) is defined by 13 basic attribute parameters, including spatial position (X, Y, Z), velocity components (U, V, W), particle diameter (D), temperature (T), and mass [37]. Among these, the vertical velocity component is set to 0 m/s, assuming all the particle leave the cup horizontally into the granulator.

Table 11 lists the input properties of particles introduced at different particle inlets. Additionally, the diameter of slag particles and that of metallic iron particles are set to 0.65 mm and 1 mm, respectively, and the initial temperature of all the particles at their inlets is set to 1773 K. The solution method selected in the numerical simulations is Phase Coupled SIMPLE.

**Table 11:** Particle properties.

Particle	X(mm)	Y(mm)	Z(mm)	V(m/s)	W(m/s)	Dslag(mm)	Dmetal(mm)
1	42.85	0.7	−8.6	−0.29	−3.29	0.65	1
2	42.85	2.2	−8.3	−0.85	−3.19	0.65	1
3	42.85	3.6	−7.8	−1.39	−2.99	0.65	1
4	42.85	4.9	−7.0	−1.89	−2.70	0.65	1
5	42.85	6.1	−6.1	−2.33	−2.33	0.65	1
6	42.85	7.0	−4.9	−2.70	−1.89	0.65	1
7	42.85	7.8	−3.6	−2.99	−1.39	0.65	1
8	42.85	8.3	−2.2	−3.19	−0.85	0.65	1
9	42.85	8.6	−0.7	−3.29	−0.29	0.65	1
10	42.85	−8.6	−0.7	−3.29	0.29	0.65	1
11	42.85	−8.3	−2.2	−3.19	0.85	0.65	1
12	42.85	−7.8	−3.6	−2.99	1.39	0.65	1
13	42.85	−7.0	−4.9	−2.70	1.89	0.65	1
14	42.85	−6.1	−6.1	−2.33	2.33	0.65	1
15	42.85	−4.9	−7.0	−1.89	2.70	0.65	1
16	42.85	−3.6	−7.8	−1.39	2.99	0.65	1
17	42.85	−2.2	−8.3	−0.85	3.19	0.65	1
18	42.85	−0.7	−8.6	−0.29	3.29	0.65	1
19	42.85	−0.7	8.6	0.29	3.29	0.65	1
20	42.85	−2.2	8.3	0.85	3.19	0.65	1
21	42.85	−3.6	7.8	1.39	2.99	0.65	1
22	42.85	−4.9	7.0	1.89	2.70	0.65	1
23	42.85	−6.1	6.1	2.33	2.33	0.65	1
24	42.85	−7.0	4.9	2.70	1.89	0.65	1
25	42.85	−7.8	3.6	2.99	1.39	0.65	1
26	42.85	−8.3	2.2	3.19	0.85	0.65	1
27	42.85	−8.6	0.7	3.29	0.29	0.65	1
28	42.85	8.6	0.7	3.29	−0.29	0.65	1
29	42.85	8.3	2.2	3.19	−0.85	0.65	1
30	42.85	7.8	3.6	2.99	−1.39	0.65	1
31	42.85	7.0	4.9	2.70	−1.89	0.65	1
32	42.85	6.1	6.1	2.33	−2.33	0.65	1
33	42.85	4.9	7.0	1.89	−2.70	0.65	1
34	42.85	3.6	7.8	1.39	−2.99	0.65	1
35	42.85	2.2	8.3	0.85	−3.19	0.65	1
36	42.85	0.7	8.6	0.29	−3.29	0.65	1

## 5. Numerical simulation results on experimental countercurrent cyclone heat exchanger

### 5.1. Grid independence check and model validation

**Fig 8:**
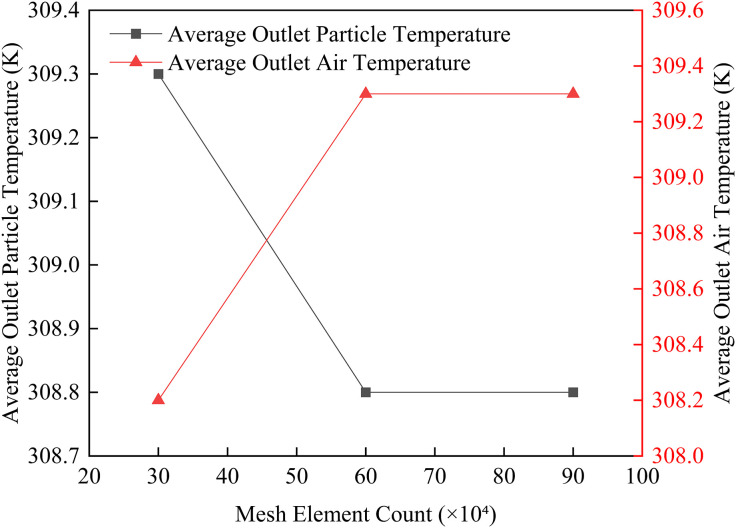
Grid independence verification.

Ansys Workbench meshing software was used for generating the grid in the computation domain of the numerical model. The mesh size in the granulation chamber region was controlled within the range from 2.5 mm to 6.5 mm, while that in the swirl channel region of the cyclone heat exchange chamber was set to 5 mm. To determine the optimal mesh density, three different mesh quantities were generated using the same method, and the air and particle outlet temperatures obtained from simulations based on these meshes under identical operating conditions were analyzed and compared. As shown in Fig 8, the results differed significantly between the 300,000 and 600,000 mesh elements, while the results were relatively close between the 600,000 and 900,000 mesh elements. Therefore, a mesh with 600,000 elements was used in the numerical model for all the subsequent simulations.

**Fig 9:**
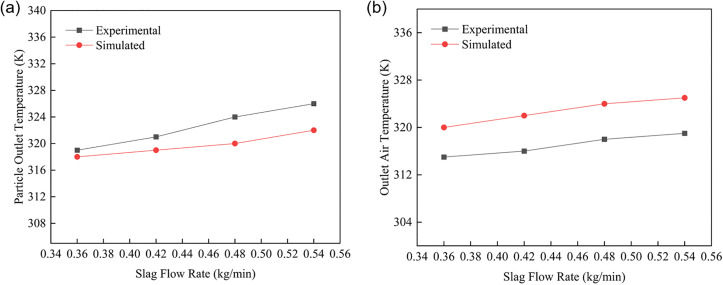
Comparison of simulated and experimental results of the effect of particle and air flow rates on their heat exchange performance. (a)Comparison of outlet particle temperature (b) Comparison of outlet air temperature.

Fig 9 presents a comparison between the measured and predicted values of particle and air outlet temperatures under different flow rate conditions. The overall trend indicates a good agreement between the numerical simulation and experimental data: as the air and particle flow rates increased synchronously, both the particle and the air outlet temperatures increased. However, within the particle flow rate range of 0.36–0.54 kg/min, the measured values of particle outlet temperature were slightly higher than the predicted ones, whereas the measured air outlet temperature was lower than the simulated values. The discrepancies primarily stem from factors such as the idealized assumptions adopted in the simulation and the measurement accuracy of the experiments as well as the uncertain amount of unavoidable heat loss to the environment in conducting the experiments. Despite some deviations, the model accurately reflects the enhancement effect of increasing air and particle flow rates on the gas-solid heat exchange process, indicating that the numerical model and results are generally reliable.

### 5.2. Air and particle flow behavior in heat exchanger

**Fig 10:**
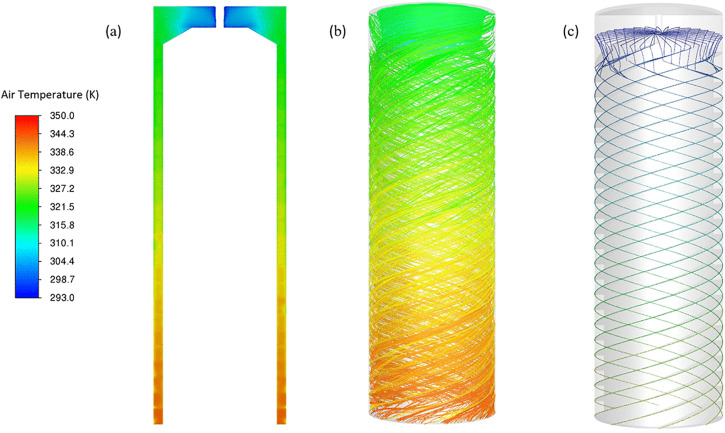
Predicted air temperature field, air flow pathlines and particle trajectories. (a) Air temperature contour field; (b) Air pathlines colored by temperature; (c) Particle trajectories colored by temperature.

Fig 10 shows the flow and distribution characteristics of air and particles within the cyclone heat exchanger simulated by the numerical model. As seen, cold air enters from the bottom inlet of the heat exchanger, confronting descending particles from the entrance at the top of the heat exchanger forming a stable countercurrent flow. Guided by the swirling vanes, the particle and air flows form a spiraling flow field inside the cyclone heat exchanger.

The air temperature contour shows that, due to the low-temperature conditions, the air temperature distribution within the heat exchanger is generally uniform, with only minor fluctuations in the inlet and outlet regions caused by particle flow disturbances, and generally no significant temperature gradient presents in the main region of the heat exchanger. This is consistent with low-temperature conditions without significant heat exchange.

The air pathline diagram clearly shows the spiral pattern of the air flow in the countercurrent swirl field. After entering the heat exchanger from the bottom inlet, under the influence of centrifugal force and the spiral flow guide vane structure, the air flow trajectories gradually transit from axially dominated pattern to a complex form coupling tangential and axial components, and rise steadily along the outer wall and finally discharge from the annular air outlet at the top of the granulation chamber. This flow structure effectively increases the contact area between the air and the particles as well as the walls.

The particle trajectory diagram reveals the motion behavior of the particles in the low-temperature flow field: Under the combined action of air drag force and their own inertia, the particles participate in the swirling motion against the countercurrent air flow. Simultaneously, driven by the centrifugal force, they migrate toward the heat exchanger outer wall, forming an annular motion path along the wall surface. Their trajectories are highly coupled with the swirling air pathlines. The motion behavior of the particles is primarily controlled by the spiral guide vanes and the air flow characteristics, which provide baseline data for subsequent heat exchange mechanism analysis and model validation under various thermal operating conditions.

### 5.3. Heat exchange performance under combined operating conditions

**Fig 11:**
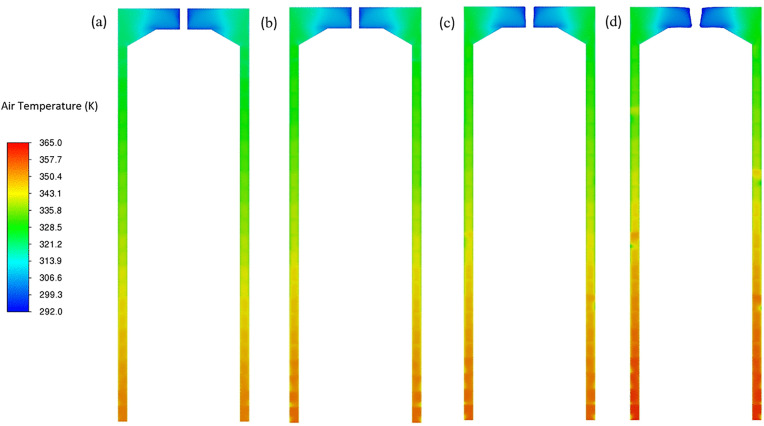
Air temperature contour plots at different air (Q) and particle (G) flow rates. (a) Q = 20 m^3^/h, G = 0.36 kg/min (b) Q = 26 m^3^/h, G = 0.42 kg/min (c) Q = 32 m^3^/h, G = 0.48 kg/min (d) Q = 38 m^3^/h, G = 0.54 kg/min.

**Fig 12:**
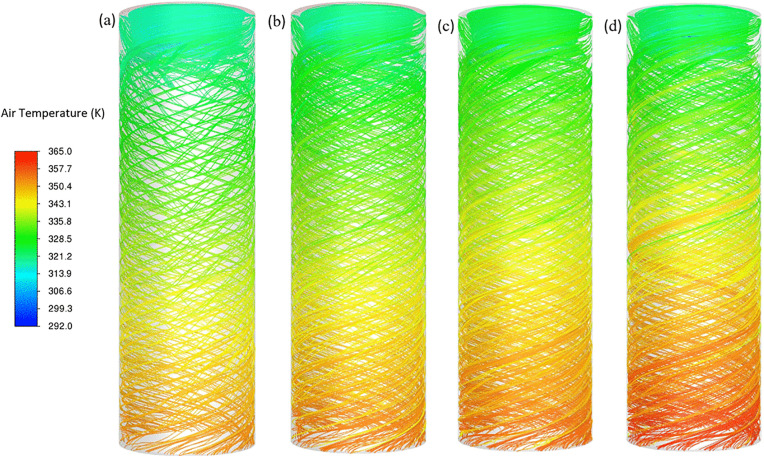
Air flow pathlines at different air (Q) and particle (G) flow rates. (a) Q = 20 m^3^/h, G = 0.36 kg/min (b) Q = 26 m^3^/h, G = 0.42 kg/min (c) Q = 32 m^3^/h, G = 0.48 kg/min (d) Q = 38 m^3^/h, G = 0.54 kg/min.

**Fig 13:**
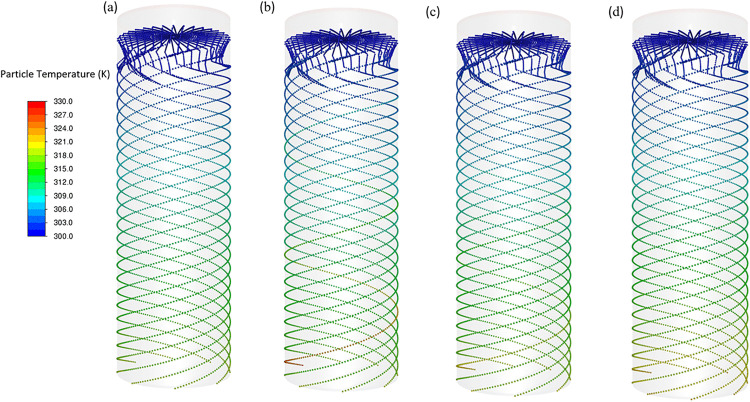
Particle trajectories at different air (Q) and particle (G) flow rates. (a) Q = 20 m^3^/h, G = 0.36 kg/min (b) Q = 26 m^3^/h, G = 0.42 kg/min (c) Q = 32 m^3^/h, G = 0.48 kg/min (d) Q = 38 m^3^/h, G = 0.54 kg/min.

Figs 11 to 13 present the air temperature contours, pathlines, and particle trajectories under combined operating conditions with synchronous increases in air and particle flow rates (denoted by symbols Q and G, respectively). To ensure validation between simulation and experimental results, the air inlet temperature was set consistently with the experimental conditions. Fig 11 shows air outlet temperature rises with synchronous increases in particle and hot air flow rates. Although higher hot air flow boosts total heat input, most heat is unabsorbed, solid slag particles have low thermal conductivity, and synchronous slag flow increase cannot fully offset their limited per-unit mass heat absorption, leaving exhaust air with significant unexchanged heat. Fig 12 and 13 further illustrate the flow patterns and swirling behaviors within the air and slag particle heat exchanger. The results indicate that an increase in the hot air flow rate leads to a rise in the outlet air temperature. Enhancing the inlet air flow rate intensifies the gas-solid convective heat transfer, whereas a higher particle flow rate improves the thermal storage capacity of the particles and consequently increases the overall heat exchange capacity of the system. Furthermore, the spatial temperature distribution is closely correlated with the particle movement trajectory.

**Fig 14:**
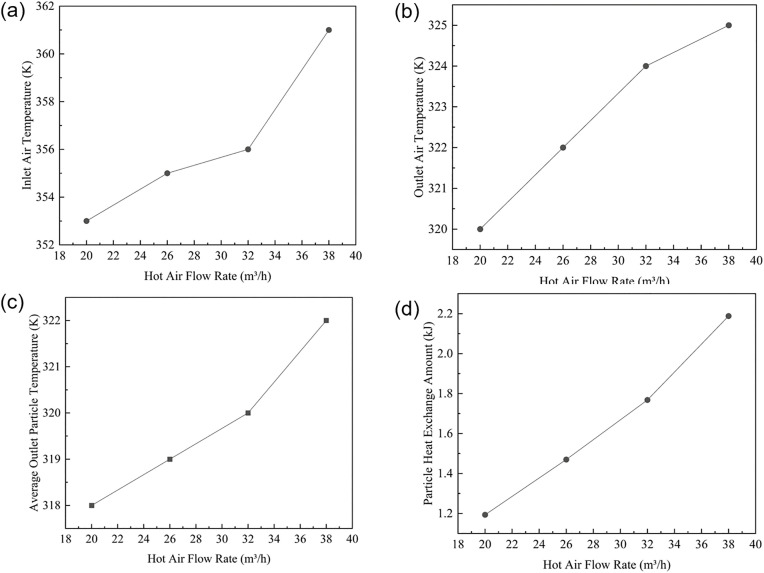
Effect of different flow rates on heat exchange performance. (a) Variation of average inlet air temperature; (b) Variation of average outlet air temperature; (c) Variation of average outlet particle temperature; (d) Variation of heat exchange amount.

Fig 14 illustrates the effect of hot air flow rates on heat exchange performance. It can be seen from this figure that the outlet particle temperature and the heat exchange amount increase nearly linearly with the synchronous increase of particle and hot air flow rates. In addition, both inlet and outlet air temperatures rise generally proportionally with the synchronous increase of particle and hot air flow rates. When the hot air flow rate increases by 6 m³/h (with corresponding increase of the particle flow rate by 0.06 kg/min), the average outlet air temperature is lifted by 2.0–2.3 K, the particle outlet temperature by 1.0–2.0 K, and the heat exchange amount by 0.277–0.420 kJ.

### 5.4. Heat Exchange Performance of Slag-Metal Particle Mixture under Combined Operating Conditions

**Fig 15:**
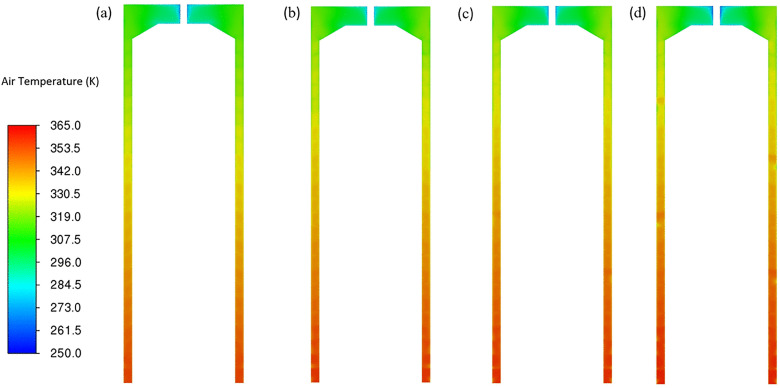
Air temperature contour plots at different air (Q) and particle (G) flow rates. (a) Q = 20 m^3^/h, G = 0.36 kg/min (b) Q = 26 m^3^/h, G = 0.42 kg/min (c) Q = 32 m^3^/h, G = 0.48 kg/min (d) Q = 38 m^3^/h, G = 0.54 kg/min.

**Fig 16:**
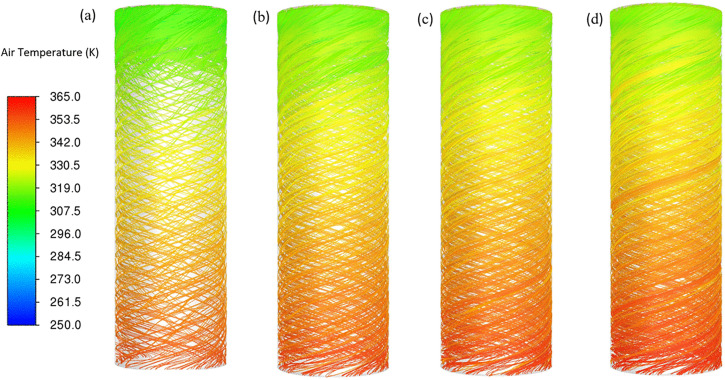
Air flow pathlines at different air (Q) and particle (G) flow rates. (a) Q = 20 m^3^/h, G = 0.36 kg/min (b) Q = 26 m^3^/h, G = 0.42 kg/min (c) Q = 32 m^3^/h, G = 0.48 kg/min (d) Q = 38 m^3^/h, G = 0.54 kg/min.

**Fig 17:**
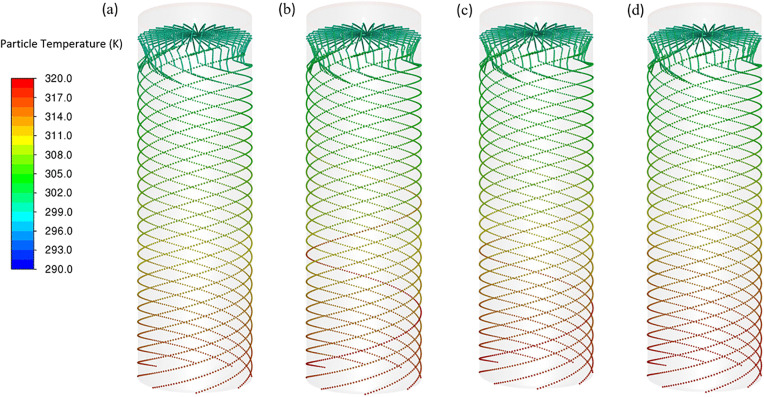
Slag particle trajectories at different air (Q) and particle (G) flow rates. (a) Q = 20 m^3^/h, G = 0.36 kg/min (b) Q = 26 m^3^/h, G = 0.42 kg/min (c) Q = 32 m^3^/h, G = 0.48 kg/min (d) Q = 38 m^3^/h, G = 0.54 kg/min.

**Fig 18:**
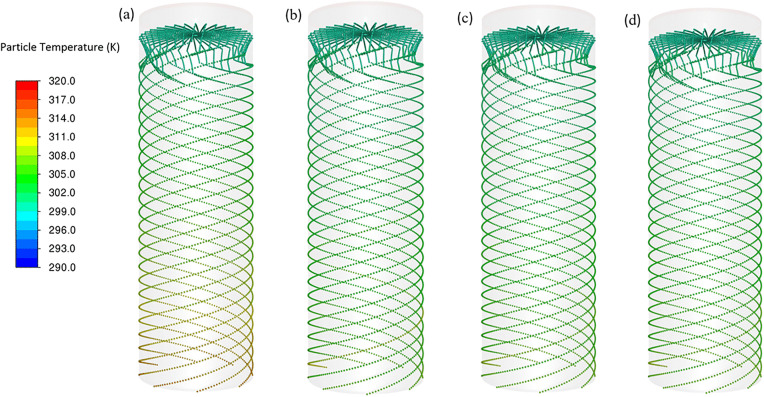
Metal particle trajectories at different air (Q) and particle (G) flow rates. (a) Q = 20 m^3^/h, G = 0.36 kg/min (b) Q = 26 m^3^/h, G = 0.42 kg/min (c) Q = 32 m^3^/h, G = 0.48 kg/min (d) Q = 38 m^3^/h, G = 0.54 kg/min.

When dealing with centrifugal granulation of molten steel slag that normally contains a certain amount metallic iron (5% to 10% by weight), mixtures of slag particles and metallic iron particles are produced. In such cases, it is interesting to the numerical model to examine the heat exchange performance of the countercurrent cyclone heat exchanger under various operating conditions to see the effect of introducing different amount of metal particles into the heat exchanger on heat transfer. Figs 15 to 18 present the numerical model predicted air temperature contours, air pathlines, and slag and metal particle trajectories under the combined operating conditions. Fig 15 shows that as the slag flow rate and hot air flow rate increase synchronously, the average air outlet temperature gradually rises. Due to the increase in hot air volume, the heat input to the heat exchanger increases. However, since the particle mixture, among which metallic particles generally have lower specific heat capacity than slag particles-exhibit relatively low overall thermal conductivity and specific heat capacity, most of the hot air fails to achieve sufficient heat exchange with the particles, leading to a continuous rise in the air outlet temperature. With the increase of hot air flow rate, the corresponding slag flow rate also increases synchronously. Fig 16 shows that as the hot air flow rate increases, the air outlet temperature keeps rising and the streamline distribution becomes denser. This indicates that under the current structural conditions of the heat exchanger, increasing the hot air flow rate not only elevates the air outlet temperature but also leads to more heat being discharged with the air flow without being fully absorbed by the particles. Figs 17 and 18 show that as the particle mixture flow rate increases, the total heat transfer amount also increases significantly. The increase in inlet air volume enhances the intensity of convective heat transfer between the air flow and particles, while the increase in particle mixture flow rate further improves the overall heat exchange capacity of the device. It is worth noting that although metallic iron particles differ from slag in physical properties, their heat transfer amount still increases with particle flow rate.

**Fig 19:**
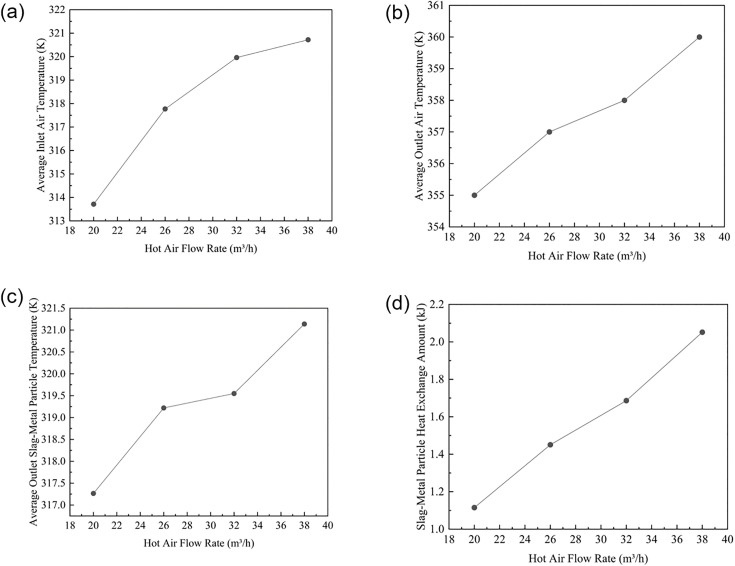
Effect of different flow rates on heat exchange performance for slag-metal particle mixtures. (a) Variation of average inlet air temperature; (b) Variation of average outlet air temperature; (c) Variation of average particle temperature; (d) Variation of particle heat exchange amount.

Fig 19 illustrates the effect of synchronously increasing particle and hot air flow rates on the heat exchange performance of slag-metal particle mixtures. It can be seen from this figure that the outlet particle temperature and the heat exchange amount increase with the synchronous increase of particle and hot air flow rates. In addition, both inlet and outlet air temperatures rise generally with the synchronous increase of particle and hot air flow rates. When the hot air flow rate is 20 m³/h (with a particle flow rate of 0.36 kg/min), the average outlet air temperature is 320 K, the particle outlet temperature is 318 K, and the total heat transfer amount is 1.115 kJ (including 1.089 kJ from slag and 0.026 kJ from metal particles).

### 5.5. Theoretical maximum heat exchange potential under adiabatic conditions

To evaluate the countercurrent cyclone heat exchanger’s theoretical upper limit under ideal operating conditions, this study conducted numerical simulations under adiabatic conditions. The operating condition neglects heat losses in the heat exchange process (such as wall heat dissipation and ambient convection) to thereby reveal the maximum heat exchange capacity determined by the heat exchanger’s own structure. Table 12 presents the simulation results for three representative optimal operating conditions: Condition 1 corresponds to the heat exchange performance of pure blast furnace slag particles; Conditions 2 and 3 correspond to the heat exchange performance of steel slag and metallic iron particle mixtures with metallic iron contents of 2.5% and 10% by weight, respectively. All operating conditions employed the constant particle flow rate (0.48 kg/min) and hot air flow rate (32 m³/h).

**Table 12:** Theoretical maximum heat exchange performance of the device under adiabatic conditions.

Particle Type	Iron Content (wt%)	Heat Absorbed by Slag (kJ)	Heat Released by Air (kJ)	Heat Exchange Efficiency (%)
Pure slag	0	271.7	339.7	80.0
Slag-metal mixture	2.5	264.9	340.8	77.7
Slag-metal mixture	10	252.4	354.0	71.3

As shown in Table 12, under ideal adiabatic conditions, the countercurrent cyclone heat exchanger exhibits excellent heat exchange performance. For pure blast furnace slag particles, the heat exchange efficiency can reach 80.0%; for slag-metal particle mixtures, however, adding metal particles essentially decrease the maximum heat exchange efficiency. The higher the metallic iron content, the lower the maximum heat exchange efficiency. when the metallic iron content in the steel slag increases from 2.5% to 10%, the maximum heat exchange efficiency falls by 6.4% from 77.7% down to 71.3%. The main reason is due to the fact that, compared with pure slag, metallic iron has a much lower thermal capacity, which leads to less absorption of heat than the slag. Therefore, it is not surprising that for the same mass flow rate of slag particles and slag-metal particle mixtures, the latter exhibits relatively lower heat exchange efficiency with extent depending on the metal content in the steel slag (i.e., the amount of slag being replaced by the metallic iron). Nevertheless, metallic iron has a much higher thermal conductivity that enhances heat transfer and thus is beneficial to heat exchange. Consequently, the heat exchange efficiency can still be maintained at a relatively high level of 71.3%. Compared with the experimental results under heat loss conditions (the optimal efficiency for pure slag is 52.0%, and for slag-metal particle mixtures is 47.8%), the heat exchange efficiency under adiabatic conditions is significantly improved, indicating the importance of thermal insulation of the heat exchanger in actual industrial design and operations of the heat exchanger.

The above results further verify the engineering feasibility of the countercurrent cyclone heat exchanger proposed in the present work in the field of waste heat recovery from molten steel slag. Although it is difficult to achieve adiabatic conditions in practical operation, recent studies have demonstrated the effectiveness of thermal insulation measures on heat loss reduction. Bany-Ata et al. [38] numerically demonstrated that applying Al_2_O_3_ and ZnO ceramic coatings on non-insulated copper heat exchangers can reduce ambient heat losses by up to 81.4% and increase net heat transfer rates by up to 35.7%, depending on flow configuration. Lee et al. [39] established a thermal design framework for heat pipe heat exchangers in waste heat recovery applications, achieving a thermal effectiveness of 78% and predictive accuracy within ±6.2% of measured energy recovery rates. Therefore, while maximum heat exchange efficiency serves as a theoretical upper bound, implementing effective insulation measures-such as ceramic coatings, proper thermal insulation of external walls, or flow enhancement devices-can enable actual heat exchange efficiency to approach 80–90% of the adiabatic conditional value. For the present countercurrent cyclone heat exchanger, thermal insulation of the external walls combined with optimized flow distribution is recommended to minimize heat losses and enhance practical waste heat recovery performance.

It should be noted that the numerical simulations in this study are steady‑state, which do not directly resolve particle residence time or its distribution. Therefore, this work focuses on the analysis of particle spatial distribution and its influence on heat exchange efficiency.

## 6. Conclusions

This study systematically analyzed the flow behavior and heat exchange characteristics of slag particles and mixtures of slag and metal particles with air in a countercurrent cyclone heat exchanger by using a combined approach of low-temperature experiments and numerical simulations. The main conclusions are drawn as follows.

1) The countercurrent cyclone heat exchanger exhibits stable heat exchange efficiency (45−50% for pure slag, 42−45% for slag-metal mixtures) under the tested flow rate ranges (0.36 kg/min-0.54 kg/min), confirming the robustness of the heat‑balance‑based flow matching strategy.
2) Numerical simulations revealed flow characteristics that are difficult to capture experimentally. The rising air forms a spiraling flow field and the particles, while descend from the top entrance to the bottom of the heat exchanger, gradually migrate toward the outer wall under the coupled actions by drag and centrifugal forces, distributing in a spiraling pattern.
3) Under adiabatic conditions, theoretical maximum heat exchange efficiencies of the countercurrent cyclone heat exchanger can be achieved reaching 80.0% for pure blast furnace slag particles and 77.7% for slag particles mixed with 2.5% metallic iron particles (particle flow rate is 0.48 kg/min, hot air flow rate is 32 m³/h, metallic iron content is 2.5 wt%, slag particle size is 0.6 mm, and metallic iron particle size is 1 mm). The latter result is useful for guiding the waste heat recovery from steel slag by using the countercurrent cyclone heat exchanger. In addition, these results are significantly higher than those obtained from experiments that entail heat losses, signaling the importance of thermal insulation in actual industrial applications.

## Supporting information

S1 Data Raw data. (XLSX)
